# Automated experimentation in ecological networks

**DOI:** 10.1186/1759-4499-3-1

**Published:** 2011-05-09

**Authors:** Miguel Lurgi, David Robertson

**Affiliations:** 1 School of Informatics, University of Edinburgh. 10 Crichton Street, Edinburgh, UK

## Abstract

**Background:**

In ecological networks, natural communities are studied from a complex systems perspective by representing interactions among species within them in the form of a graph, which is in turn analysed using mathematical tools. Topological features encountered in complex networks have been proved to provide the systems they represent with interesting attributes such as robustness and stability, which in ecological systems translates into the ability of communities to resist perturbations of different kinds. A focus of research in community ecology is on understanding the mechanisms by which these complex networks of interactions among species in a community arise. We employ an agent-based approach to model ecological processes operating at the species' interaction level for the study of the emergence of organisation in ecological networks.

**Results:**

We have designed protocols of interaction among agents in a multi-agent system based on ecological processes occurring at the interaction level between species in plant-animal mutualistic communities. Interaction models for agents coordination thus engineered facilitate the emergence of network features such as those found in ecological networks of interacting species, in our artificial societies of agents.

**Conclusions:**

Agent based models developed in this way facilitate the automation of the design an execution of simulation experiments that allow for the exploration of diverse behavioural mechanisms believed to be responsible for community organisation in ecological communities. This automated way of conducting experiments empowers the study of ecological networks by exploiting the expressive power of interaction models specification in agent systems.

## Background

### Complex Systems

Complex systems of interacting entities are ubiquitous in nature and the artificial world, ranging from networks of interacting computers in cyberspace, different types of transportation networks such as airports and the links connecting them, power grids that provide cities with electricity, human interactions of different kinds such as researchers and their collaborations; to the highly organised webs of interactions that we find in biological systems such as protein, gene, cell, and neural networks, and the relationships found in real communities in a given ecosystem, where the interacting species, each of them with its own genetic, phylogenetic, life-history, and evolutionary background come together in a particular habitat and form complex but organised collections of interacting entities.

All of these are examples of complex systems, where the system-level organisation emerges as a consequence of the coupled interactions amongst their component parts rather than being exerted by some control mechanism operating at the level of the system. The behaviour and organisation of such systems are then, not easily inferred by only looking at the interacting components in the system but it is caused by the set of complex and intricate relationships that are realised between them.

Network theory has facilitated the study of complex systems in a large number of areas [1,2], where the configuration of the interactions among entities are of utmost importance for the stability and long term behaviour of this type of systems. By using mathematical properties obtained from the graph representing the network of connections between entities in complex systems, important features of their overall organisation, such as their robustness to the failure of nodes, or the information propagation speed; properties which in turn may provide a better understanding of the relationship between the complexity and stability of the systems being analysed.

### Ecological Interactions

Animal behaviour and interactions comprise a subject of study that has historically received much attention in the field of ecology [3-6], not only because of the wide set of different types of interactions that can occur among species but also because these interactions are believed to account for the huge biodiversity encountered in our planet and more specifically for the organisation of communities within the very dissimilar collection of ecosystems found on Earth.

Different classifications have been attempted for categorising the whole range of interactions encountered between species in nature, and roughly, interactions fall into two main categories: antagonistic and mutualistic interactions. In this work we are mainly interested in mutualistic interactions, in which both of the interacting partners/species benefit from it, and how networks of relationships between mutualistic species emerge in nature.

**Figure 1:**
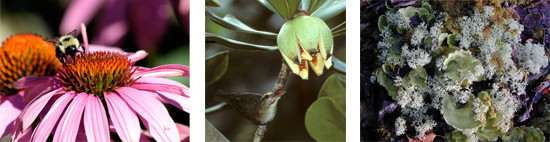
**Examples of mutualistic relationships in nature**. From left to right: plant-pollinator interaction between a bee and a flower, plant-frugivore interaction between a bird and a fleshy fruit plant, and mutualistic association between fungi and algae (i.e. a lichen).

Mutualistic relationships are ubiquitous in nature (Figure 1), ranging from the interactions between plants and their animal pollinators or seed dispersers, without which life on Earth could seldom be imagined, to the complex association between fungi and algae that form lichens. All these interactions have arisen in nature because they represent an important advantage for the individuals taking part on them, and moreover, in some cases, one or both of the interacting partners would not be able to survive outside the interaction.

#### Ecological Networks

Charles Darwin described the intricate network of relationships between species in a natural community using the metaphor of an "entangled bank", in which he depicted a typical natural scene of a group of different species living together and interacting. In this example scene we can see represented the web of complex relations by which species in natural ecosystems are bound together [7].

A focus of research in ecology is based on the characterisation of different kinds of interactions that are observed amongst individuals within ecological communities. The study of the patterns and structure of these interactions and their implications for community organisation and persistence has been tackled through the application of concepts borrowed from the mathematical field of network theory, giving way to the ecological discipline that has come to be known as ecological networks.

**Figure 2:**
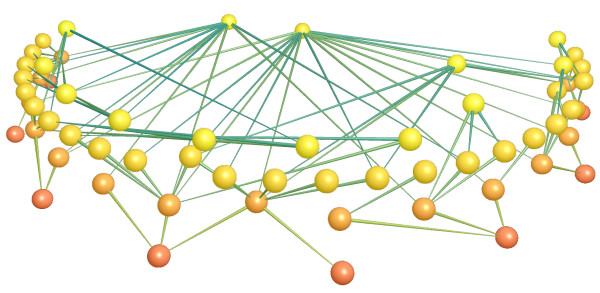
**Example of a complex food web in a real community (grassland in the United Kingdom)**. Nodes and edges (in the graph) represent species and trophic interactions among them respectively. (Image produced with FoodWeb3D, written by R.J. Williams and provided by the Pacific Ecoinformatics and Computational Ecology Lab http://www.foodwebs.org).

In ecological networks the interactions between species in natural communities are represented in the form of a graph, where vertices and edges represent species and their relationships respectively. Figure 2 shows an example of a food web, a kind of ecological network in which the edges between species represent trophic relations, from a real community (a grassland in the United Kingdom). The representation of species in a community and their interactions thus facilitates the analyses of this kind of complex system. Ecological network research is concerned with the analysis of the relationships amongst species in an ecosystem from a network perspective, in order to determine community level features such as those described above for complex systems. These analyses have allowed scientists to better understand the organisational dynamics of the ecosystems represented by these entangled networks of interactions by establishing links between the dynamics occurring at the individual and species level to the structure of the network representing their relationships [8].

Studies of this kind performed on natural communities, have shown that these systems are generally characterised by small world patterns [9,10], contributing in this way to fast propagation of the information across the networks; truncated power law degree distributions [11], a feature characteristic of scale-free networks, with a small number of nodes possessing degrees greatly exceeding the average (usually referenced as "*hubs*") that are believed to be the strength and, at the same time, the weakness of this kind of networks; and the importance of weak interactions for the maintenance of the overall community [12]. As an example of some of these features, in Figure 2 we can see how no species is distant from the most connected ones (small world) and it can also be appreciated a set of species (the two nodes on the top centre of the figure) which degree exceeds considerably that of the rest of species, giving this network a scale-free character.

In addition to these features, other properties believed to be important for the characterisation of interaction networks in natural communities such as: species richness, connectance, link distribution frequencies, among others are also commonly studied (e.g. [13-15]).

In this paper we are interested in the study of plausible mechanisms, occurring at the interaction level between species in ecological communities, for the emergence of important system-level patterns often encountered in ecological networks and that provide these systems with the properties introduced above. We focus on the interactions and patterns seen in mutualistic communities, which are studied through the analysis of mutualistic networks.

#### Mutualistic Networks

Within ecological networks, a specific area of research is that devoted to the study of communities from a mutualistic perspective. Species and their mutualistic relationships can be represented as graphs in the way described above in order to obtain the mutualistic network of interactions for a particular community, which can in turn help us study the effects of these mutualistic interactions on the organisation of the community.

Studies on mutualistic networks have demonstrated that these kind of networks share some of the patterns and features described above with other kinds of ecological networks [16,17], as for example their heterogeneity or scale-free character, possessing a small proportion of species that are more connected than expected by chance while the majority of them exhibit a low degree; but at the same time are different in many respects, exhibiting properties that are characteristic of these kind of networks: with generalist species interacting with subsets of the species with which more generalist species interact (i.e. they are nested) [18], and being pervasively asymmetric regarding the links between species [19].

An important distinction to be made, because it is going to determine the shape of the networks of interactions between agents in our agent-based model, is the fact that mutualistic networks are represented by bipartite graphs, since the species are separated into two sets of distinct entities: the hosts and the mutualists or, specifically for the case of mutualistic relationships between plants and their free living animal pollinators, the hosts and the visitors. This important property is also what allow us to obtain measurements that are exclusive of these kind of networks, like for example their nestedness (i.e. the degree to which the interactions of specialist species are well-defined subsets of the interactions of more generalist species).

**Figure 3:**
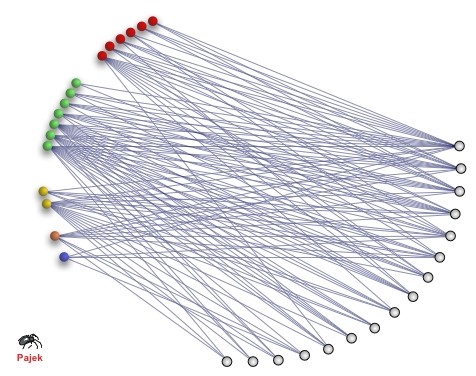
**Example of a mutualistic network of a real community**. Nodes and edges represent species and their mutualistic relationships respectively. (Figure provided by P. Jordano. Reproduced with permission.)

Figure 3 shows an example of a mutualistic network, where it is possible to see how the species are arranged in clearly distinct groups, forming a bipartite graph, where species only interact with species on the other side of the graph. This is easily understood when we imagine a community of flowering plants and their animal pollinators (e.g. insects, birds); in such cases the mutualistic relationship is based around the pollination service provided by the animals present in the community, therefore different animal species interact with plant species but they do not interact with other animal species in this sense (they might interact in other ways, but that are not relevant for the mutualistic relationship subject of study), similarly, plants do not pollinate other plant species, and hence, there are no links between nodes representing plant species.

The mechanisms by which ecological networks are formed and maintained across landscapes and through time are currently not completely understood, and nowadays researchers within the field of ecology are focusing efforts on this topic in order to be able to predict the effects that disturbances may have on community organisation, biodiversity maintenance, and ultimately ecosystems function.

This is in fact a complex research agenda because, as we have seen, ecological networks are complex systems, exhibiting many of the features studied within the field of the complex sciences. As in any other area of this research field, natural communities are systems composed of many interacting entities that are under constant coevolutionary and adaptive change and which are dynamically self-organised; to put it simply, they are systems of complex interacting parts in which the system-level properties and behaviour are very difficult to explain by only looking at their component parts. In ecology the problem is further complicated by the fact that organisms in communities are themselves complex systems, and only to begin to fully understand these component parts of the whole system is a very difficult task in itself.

Within the field of mutualistic networks, however, some advances have been made on this direction, and some processes have been identified as potential explanatory mechanisms for the patterns encountered at the system level in this particular type of ecological networks. When studying natural communities it is common to search for answers about system level properties in the biological and ecological processes going on at the level of the individuals forming those communities and the relations amongst them, and it has been argued that such mechanisms might be responsible for the features seen in these complex mutualistic associations of species [20].

We are particularly interested in those mechanisms because in this work we have taken inspiration from some of the processes characteristic of mutualistic interactions, such as for example: *trait matching, habitat *occupation (i.e. spatial distribution), the formation of *meta-communities*; to define protocols of interactions among agents in an agent based model that will allow us to study the extent to which these and other mechanisms might be behind the patterns encountered in mutualistic communities in an automated manner.

By specifying protocols of interactions between agents in our model, we want to facilitate the experimentation, based on computer simulations, on diverse processes that might be thought as potential mechanisms for the explanation of organisational patterns encountered in this kind of natural communities.

### Models for the Study of Ecological Networks

As mentioned above, several studies have focused on the problem of understanding the processes and mechanisms that might be behind the emergence of the properties found in ecological networks in general and mutualistic networks in particular. In [20] the authors review a series of theorised mechanisms that have been proposed in recent years within the field of ecology, as possible causes for the appearance of these patterns in plant-animal mutualistic networks. It is from these ideas that we depart in this work. The study of these and other mechanisms and their consequences however, is difficult to achieve in real ecosystems, not only because of the complexities of the experimental settings needed in order to be able to analyse a whole community and the interactions within it, but also because of the large timescales at which changes in the composition and configuration of communities might happen. Modelling these systems have been then an useful an important approach taken by many scientists studying the origins of patterns in ecological networks.

These studies usually focus on particular mechanisms that the authors favour as important processes behind the systems' patterns and behaviour. In [21], Williams and Martínez propose the nichemodel, which based on the hypothesised dietary niche of the species composing the system performs well in obtaining some of the common features encountered in ecological networks. Another interesting approach have been proposed by Petchey et al. [22]; in this model, the authors incorporate body size and foraging behaviour, grounded in metabolic factors, into the framework of food web structure analysis.

Approaches like the ones mentioned above are grounded on the behavioural ecology and phenotypic features of species. Another trend in the study of these ecological systems considers evolution to be an important mechanism behind their organisation. Drossel and McKane [23,24] developed a food web model with evolution which builds on these ideas. In their model, population dynamics in the system are governed by differential equations, in which functional responses, the terms that determine the type of interactions between species, are determined by a series of attributes of each species. These attributes are subject to variation and selection.

The models introduced above are the most closely related approaches found in the literature to our own work, since they incorporate ideas of animal behaviour similar to those studied in this paper for the representation of interactions between agents in our model. Nonetheless, many other approximations have been proposed (see [8] for a summary) focusing on other aspects of the origin of ecological networks.

In addition to the fact that we focus on different aspects of the behaviour of species than those considered in previous models, our approximation goes beyond the studies presented above at least in two other respects: (i) it allows for the specification of interaction mechanisms between agents/species in a straightforward manner, which facilitates the work of the researcher because it makes easier the translation of ecological mechanisms into the model; this way of specifying interactions (ii) makes the model easily extendable, facilitating the incorporation of new processes and mechanisms into the experimental framework. Both of these features support the experimentation in an automated manner.

### Interaction Centred Agents Systems

In the field of multi-agent systems, different approaches for communication between entities living in this kind of distributed environments exist, and recently, approximations based on the specification of protocols of interactions for agent coordination and communication have been proved to be useful in large systems because of their scalability and compactness [25].

Apart from the properties mentioned above, which are of relevance mainly to the multi-agent community, we are interested in methods of this kind for communication in multi-agent systems because they are mainly based on the definition and use of protocols for coordinating agents taking part in a given interaction, which represents an easy and straightforward way of translating ecological concepts and processes that are of interest for the study of species interactions.

In the remainder of this section we present one of the tools available within the multi-agent systems community for implementing agent based systems with the characteristics described above. We explain the main features of the system and the coordination language employed for agents' communication within it, which allows us to present the reasons why we have chosen this system for implementing our agent based model.

#### Lightweight Coordination Calculus

Lightweight Coordination Calculus (LCC) [26] is a process calculus that has been proposed as a language for specifying interactions between heterogeneous electronic entities operating in peer-to-peer (P2P) environments. Apart from allowing a distributed coordination of the interactions amongst artificial agents through protocols, it is a lightweight language that provides flexibility and clearness for the specification of the interactions.

The use of LCC for the development of our agent based model allows us to program in an interaction oriented manner, where we focus on a declarative specification of the coordination protocol, the execution of which is totally independent of the design of the agents taking part within it.

This is advantageous from a behavioural ecology point of view because the system's designer need only to focus on the mechanisms and processes occurring at the interaction level between species in natural communities when specifying the interactions between the agents in the model.

#### LCC Syntax

The syntax of LCC, as well as the specification of its expansion engine and framework, have been detailed in a series of papers [26-28] and we are not going into all of its details here but we summarise the important parts to bear in mind about interaction models specification using this language for the definition of the interaction protocols in our agents system.

**Figure 4:**
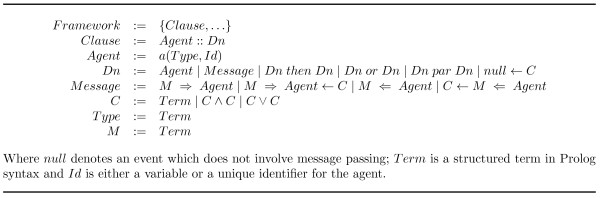
**Syntax of LCC dialogue framework**.

In Figure 4, taken from [28], the syntax definition of the LCC is presented, including an explanation of the language itself and the *framework*. Any protocol thus specified consists of a set of agent clauses, as specified by the definition of the Framework; and will contain at least two of them, because in any given interaction there must be at least two roles in order for it to happen. These interactions are defined from the perspective of the participating agent roles, such that in any given interaction at least one participant taking each role specified by the protocol, must be present in order for the framework to be successfully completed. Each clause is defined by a series of communicative actions (i.e. messages) that are performed by the agent adopting the role defined by the given clause. These clauses are essentially the definition of the agents, or more precisely, the specification of the actions to be undertaken by any agent in the system assuming a particular role as defined by a clause.

A role defined within a protocol in this way is meant to represent the communicative activity for a group of agents (any agent able to fulfil the role) instead of individuals. Clauses' definitions consist then of a set of operations *Dn *in Figure 4. These operations will be used for the specification of the protocol: *null *denotes an event which does not involve message passing; control flow operations such as: *then *, *or *, *par *, are logical connectives used to determine whether actions must be executed in sequence, only one of them should be executed (choice), or they have to be executed in parallel, respectively; the operator ← denotes logical implication and is used to define constraints that determine agents' obligations for each role in the protocol, with preconditions appearing on the right of the ←, and postconditions on its left; *M *⇒ *Agent *denotes that a message, M, is sent out to agent "*Agent*"; and *M *⇐ *Agent *denotes that a message, *M*, from agent "*Agent*" is received.

Another advantage of using LCC is related to its compactness regarding the definition of interactions among species in our ecological model, which permits simple and powerful mechanisms for analysis and deployment. This feature let us infer in an efficient manner the outcome of particular interactions under certain circumstances without compromising the level of detail that we intend to achieve when defining those interactions, which is useful when considering different species interaction mechanisms and the behaviour they might produce.

#### OpenKnowledge

For the implementation of our agent based model of interacting species we employ the OpenKnowledge system [29,30], which allows us to use protocols of interactions of the kind described above for the formalisation and description of the interactions amongst entities in a digital system in general and agents in a multi-agent system in particular.

The OpenKnowledge system is a P2P based system that makes use of LCC for the specification of models of interaction among autonomous agents which communicate through a P2P network. This open source system provides an interaction centred approach for knowledge sharing and agents communication that facilitates the engineering of intelligent systems using the multi-agent systems paradigm for software engineering.

The details of the architecture and how the system works are explained in [29] and [30], however, there are two concepts that are important for the realisation of the interaction among agents/individuals within the system/ecosystem, and that will be useful for us when defining the agents and their interactions in the model:

1. Interaction Models (IMs): Interaction models are the formal specifications of the protocols to be followed by any pair of agents involved in an interaction, which are specified in a language devised for this purpose, in our case LCC. IMs contain the definition of roles and the interactions between them.

2. OpenKnowledge Components (OKCs): OKCs are components within the OpenKnowledge framework that implement the roles specified in IMs. An OKC implemented for fulfilling a particular role in an IM must possess the implementation of all of the constraints required for that role (i.e. it must be able to successfully complete any interaction based on the IM). Is within the OKCs where we must specify the behaviour to be followed by each species in our ecosystem model in respect to the IMs they are going to follow for their interactions.

Through the specification of IMs using LCC as a protocol specification language and the implementation of OKCs, we describe agents and their interactions inside an agent based ecological model. We want to employ a model developed in this way for the study of the emergence of interesting organisational patterns in natural communities and the likely ecological mechanisms and processes that are behind them. This model aims thus to facilitate the experimentation of mechanisms that are believed to enable the system level organisation of natural communities, in an automated manner; by specifying interaction models between simulated species (agents in the agent-based system) based on ecological theory.

## Results and Discussion

Based on the ecological ideas presented so far and making use of LCC and the OpenKnowledge framework for the development and deployment of a multi-agent based simulation platform, we have designed and implemented ecological protocols of interaction for defining the relationships among agents in simulated ecosystems. These protocols are mainly focused on the ecological processes occurring at the interaction level between species in plant-animal mutualistic communities (see Methods).

We have found that interaction models for agents coordination thus engineered facilitate the emergence of network features such as those seen in ecological networks of interacting species in real communities, in our artificial societies of agents. In this section we present these results and we discuss how an ecological model of this kind can be used to study the ecological mechanisms behind the emergence of the network patterns we see in our agent societies and which are characteristic organisational features of the ecological communities they represent.

Our experiments consisted of the execution of a series of independent simulation runs following the model specifications outlined in the Methods section for parameter initialisation and runs configuration. During these runs, relationships among pairs of agents arose with different strengths (the number of times an agent interacts with any other relative to the number of times it has interacted during the entire simulation) and with different configurations.

We ran one hundred simulations where the configuration and properties of the networks of interactions between agents/species where similar among them. In order to be consistent throughout this paper we have selected fifteen samples from the simulated networks obtained, because, as we will see below, this was the number of natural communities that we were able to extract from a dataset of empirically obtained mutualistic networks that were more similar in number of species to our networks, and that we will employ for the analysis of similarities between the interactions networks obtained by the model and those observed in the real world. Changing the set of selected networks for analysis would not noticeably affect the results presented.

In this section we analyse the structure and features of this set of simulated networks. Apart from the identity of the agents, which is changed in every run based on the different configurations they can be initialised with (as explained in Methods), the architecture was, in general, similar among the obtained interactions networks; presenting constant patterns that are also found in ecological networks.

**Figure 5:**
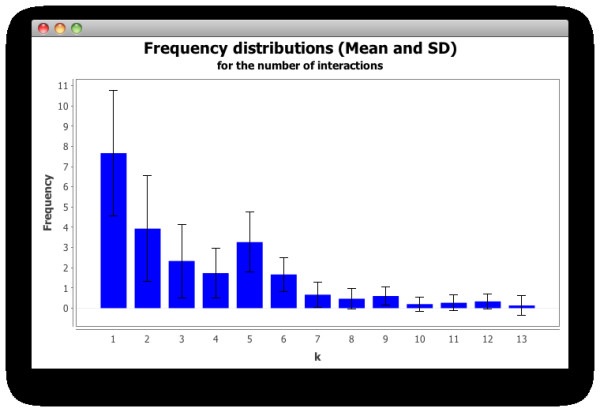
**Distributions of frequencies of nodes degrees in simulated networks**. Frequency distribution of the mean (with its standard deviation) of the number of interactions per node in fifteen of our simulated networks.

The mean and its standard deviation for the number of interactions occurring per node are plotted in Figure 5 as the distribution of the frequency of each number of interactions among the nodes in our networks. Although the standard deviation from the mean value is high for small degree values, we can see in this plot that the frequency of the number of interactions among nodes is highly biased towards small values and that few nodes among all the networks possess high degrees (i.e. are highly connected). It is a common pattern in our simulated networks then, that the majority of nodes possess low degrees, or interact with only one or a few other nodes in the network, and only a small fraction of agents are well connected to others in the simulated ecosystem.

As we shall see, these are characteristic features that are believed to account for interesting properties of a particular kind of complex networks, which have come to be known as scale-free networks, and are important features observed in the mutualistic natural communities we are interested in analysing.

**Figure 6:**
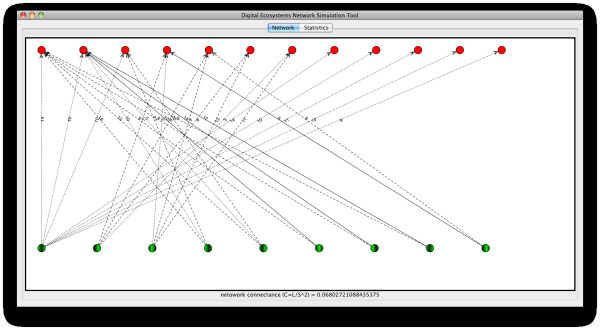
**Example of the output of a simulation run performed using the model described**. A network of interactions between artificial species in a simulated ecosystem. Host and visitor agents are represented by green and red nodes respectively. The thickness of the arcs represent the relative strength of that interaction relative to other coming from the same host species, while numbers on them indicate the number of times that particular interaction has been observed.

Figure 6 shows an example, taken from one of the runs in our experiments, where the relationships among agents in our system are represented in a fashion similar to networks of interacting species described in the field of ecology (as we saw the Background section).

As explained in Methods, we have defined two roles for agents to take in our simulations, in resemblance to the actors taking part in plant-animal mutualistic interactions in nature: the "host" and the "visitor". In our network representations we have represented "host" and "visitor" agents/species as green and red nodes in the graph respectively. Relationships between agents are represented by arcs (directed edges), where the direction of an arc represents the direction of the energy flow in the ecological system while its thickness represents its relative strength with respect to the other connections that depart from the same host (green node). Numbers on edges represent the number of times that particular interaction was observed in the simulation (i.e. the number of times the pair of agents linked by that arc completed a successful interaction). A link (arc) is thus generated between two agents whenever an interaction is successfully completed amongst them.

By representing the relationships between the agents in our artificial ecosystems in this way we are able to extract features, obtain descriptors, and perform analyses over the resulting network based on methods borrowed from network theory.

**Figure 7:**
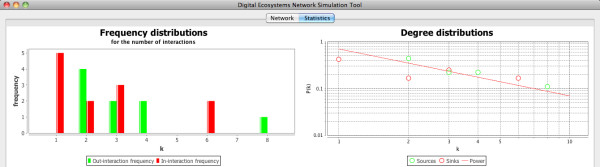
**Plots corresponding to the network displayed in figure 6**. The plots show the frequency distribution of the number of interactions and the distribution of degrees among the nodes on the network.

As introduced above (Figure 5), we obtained networks that display a scale-free structure. The plots displayed in Figure 7 show the data from the network in Figure 6, where it can be observed that the majority of nodes in this network have small degree (≤ 2), while a low fraction of them are highly connected, showing a distribution of the frequency of interactions (left plot) biased towards low values (1 and 2 interactions), and a distribution of degrees (right plot) with a decreasing slope in a fitted power law. Additionally, small-world properties are found in our networks: with short paths between any two nodes. Properties of the kind mentioned above, which are encountered in the networks of interactions among our agents in the simulated communities, are common patterns also found in different kinds of complex networks in nature and the artificial world [1], and which differ significantly from the structure that we would expect from a randomly assembled network.

Another property seen in our networks, which is related to their scale-free character, is the preferential attachment displayed by visitor agents with low degrees (e.g. the five red nodes on the top right corner of Figure 6) to host agents that are highly connected. This is a common feature encountered in mutualistic networks, where specialist species are more likely to interact with generalists [16]. Patterns of this kind are important in practice because, as it has been argued, they can give us information about functional properties of the communities such as: disturbances propagation speed and robustness to species loss, which in turn provide us with a better understanding of the relationship between the complexity and stability of our ecological systems.

Asymmetric specialisation (i.e. a specialist interacting with a generalist) has been found to be a pervasive feature of plant-pollinator interactions networks, and it is believed to be beneficial for the majority of species in these communities because it facilitates the avoidance of extinction risks when species are highly reciprocally specialised [19]. This is another feature arising in the network of interactions among agents in the model presented.

The features encountered in the networks of relationships amongst our artificial agents are in many ways similar to those found in real mutualistic networks, as shown above; which are patterns that differentiate random networks from self-organised complex networks of relationships. This network architecture is an emergent property of our agent based system since the only mechanisms involved in agents' interactions are those specified by the protocol of interaction presented in the Methods section. The creation of such a complex and intricate pattern of relationships is not a hardwired property of the artificial communities arising from the simulations performed, but rather the product of many different agents interacting together for achieving their respective goals (gather resources and survive).

This conclusion is important because it means that the ecological and behavioural mechanisms that we are studying and that are translated into interaction models between agents in our computer model are directly and solely responsible for the system level attributes found in the artificial communities thus enabled, becoming in this way plausible causes for the emergence of these features in the studied systems.

### Comparing with Empirical Data

In order to test the extent to which the networks of interactions found in our artificial systems are similar to mutualistic networks of interactions found in the real world (apart from the qualitative similarities introduced above), we have compared the architecture of the networks obtained from our simulations to some networks empirically collected from real communities and that have been compiled, analysed, and provided as supplementary material by Rezende et al in [31]. Although in that paper they used the networks for different kinds of analyses, the datasets provided are useful for getting an idea of the common features encountered in mutualistic networks.

Because some of the properties of interest for analysing ecological networks are scale dependent, we have selected fifteen networks from this dataset, based on the number of species composing it and that were closer to the number of agents in our simulations, for comparison against fifteen of our simulated networks. Connectance (*C *= *L*/*S*^2^), the fraction of all possible links that are realised in a network, is an important property that is commonly employed in the analysis of ecological networks and which provides information about the degree of connectivity between their nodes. We use this network measure and the Nestedness metric based on Overlap and Decreasing Fill (NODF), introduced by Almeida-Neto et al in [32], which provides a measure for community organisation in plant-animal mutualistic networks of interactions based on the overlapping diets of the species in the community studied; for comparing our simulated networks with fifteen empirically obtained plant-animal mutualistic networks. Additionally we perform qualitative comparisons between the structures obtained in one of our networks and one of the natural communities considered for this analysis.

**Figure 8:**
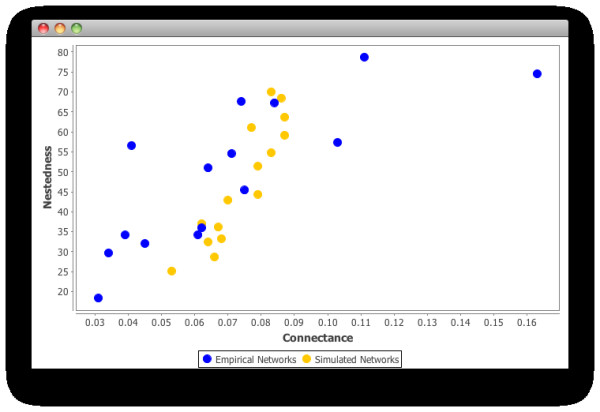
**Connectance versus NODF nestedness values in natural and simulated communities**. The values plotted are the connectance vs the NODF nestedness indexes obtained from the natural (blue dots) and simulated (yellow dots) communities presented in table 1 (see text).

In table 1 we can see the connectance and NODF values derived from our simulated networks and the selected empirical networks obtained from natural communities, and in Figure 8 we plot the NODF against the connectance values with the double purpose of analysing the behaviour of nestedness in relation to changes in the connectance of the network, and to compare these relationships in our simulated communities against their natural counterparts.

**Table 1:** Connectance and NODF index of nestedness values from the artificial and real networks.

Simulated	C (*L */*S*^2^)	NODF	Empirical	C (*L */*S*^2^)	NODF
1	0.053	25.203	SAPF	0.031	18.36
2	0.062	37.063	CACO	0.034	29.693
3	0.064	32.456	CAFR	0.039	34.166
4	0.066	28.667	SCHM	0.041	56.66
5	0.067	36.132	MOMA	0.045	32.067
6	0.068	33.333	GEN1	0.061	34.243
7	0.07	42.913	OFLO	0.062	35.961
8	0.077	61.06	BAIR	0.064	50.98
9	0.079	44.409	ESKI	0.071	54.586
10	0.079	51.404	BEEH	0.074	67.66
11	0.083	54.82	WYTH	0.075	45.411
12	0.083	70.102	KANT	0.084	67.344
13	0.086	68.528	LOPE	0.103	57.423
14	0.087	59.211	HRAT	0.111	78.756
15	0.087	63.739	FROS	0.163	74.571

The values were taken from the networks of interactions of fifteen simulated digital communities of agents and fifteen empirically obtained networks from natural communities. The names of the empirical networks are as presented in [31], and the numbers used to identify the simulated networks are only for id purposes and are not related to any feature of the network itself.

As we can see, in both types of networks there is a positive relationship between the connectance of the network and the nestedness value. This is not surprising, since the more connections a network has the more they can contribute to the nested diets observed in those networks. It is perfectly possible however, that more links could mean less nested communities; it is important to bear in mind then that in well organised communities, like the ones we consider in this work, the more connected networks are, the more nested they become.

We also can see from this plot (Figure 8) that the data points corresponding to the naturally occurring communities are more distributed through the graph, while the points representing our simulated networks are more concentrated around connectances between 0.05 and 0.09, and nestedness indexes of 25 and 70. Apart from telling us about the variability encountered in real communities in this respect, this reinforces the fact that our simulated communities, although differing in some aspects from run to run, share a similar structure and enjoy features seen in natural communities.

These data (table 1 and Figure 8) also show us that the values of connectance and nestedness obtained from our simulated communities agree with the values of these measures commonly found in natural communities, where the connectance is normally between 0.03 and 0.1, and the nestedness values are usually found between 20 and 80. In our communities, the connectance values were greater than 0.05 and lower than 0.1; similarly, the NODF values for our communities were distributed along the 25-80 range of values. Apart from showing a connectance-nestedness relation comparable to that found in natural communities, these values are also in line with what is expected to find in these, which provides more evidence for the self-organised character of the communities modelled.

We want to further explore the similarities between our agent-based model communities and natural ones; for this we have selected, from the set presented above, one of our simulated and one of the empirically observed systems to perform a one to one comparison.

Two networks were thus selected based on their similitudes not only in connectance and nestedness values but also taking into account other features, as we shall see in the following paragraphs. The networks selected were: the one represented by the number 3 in table 1, with values 0.064 and 32.456 of connectance and NODF index for nestedness respectively, for representing our simulated communities; and the OFLO natural community, with 0.062 connectance and 35.961 NODF index, as a representative of the natural communities considered.

**Figure 9:**
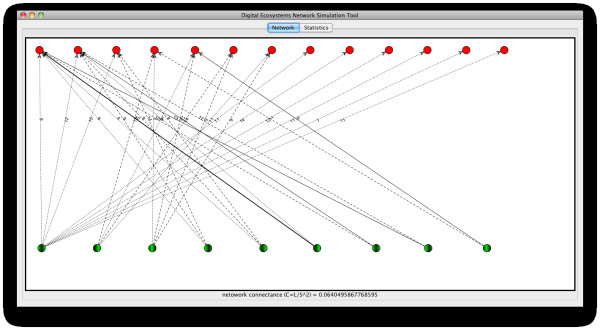
**Network representation of community number 3 in our simulations as presented in table 1**. Host and visitor agents are represented by green and red nodes respectively. The thickness of the arcs represent the relative strength of that interaction relative to other coming from the same host species, while numbers on them indicate the number of times that particular interaction has been observed.

**Figure 10:**
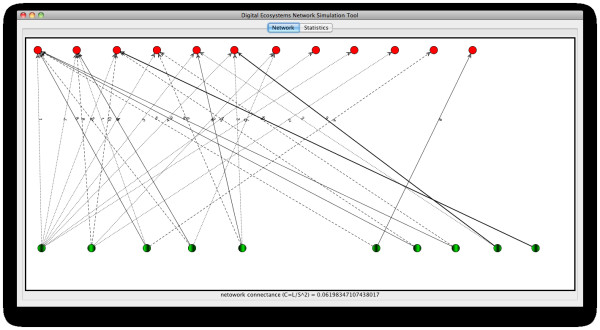
**Network representation of the OFLO natural community as introduced in table 1**. Host and visitor agents are represented by green and red nodes respectively. The thickness of the arcs represent the relative strength of that interaction relative to other coming from the same host species, while numbers on them indicate the number of times that particular interaction has been observed.

Figures 9 and 10 show the network of relationships between entities in the simulated community number 3 and the OFLO natural community, respectively.

When closely inspecting these two networks we can easily realise a number of broad similarities such as the high proportion of visitor nodes (nodes in red) possessing only one interaction and also an important fraction of host nodes (nodes in green) with two interactions or less. It also attracts our attention the presence of one highly connected host node in both of the networks (green node in the bottom left corner in each of the networks), and also on the side of the visitor species/agents (red node on the top left). These nodes act as generalists/hubs, bringing cohesion and reachability to the whole network.

Another important feature captured by looking at the network of interactions and that is shared by our natural and artificial communities is the low fraction of strong dependences among nodes (solid dark edges in the graph) and the abundance of weak dependences, which in natural communities is believed to account for the stability and resilience of these systems, since the loss of a link can be easily adjusted for by recurring to other connections in the network.

Apart from the graphical representation of the interactions network, our simulation tool allows us to analyse certain properties derived from its structure and that can be employed to deepen our comparison among the selected networks.

**Figure 11:**
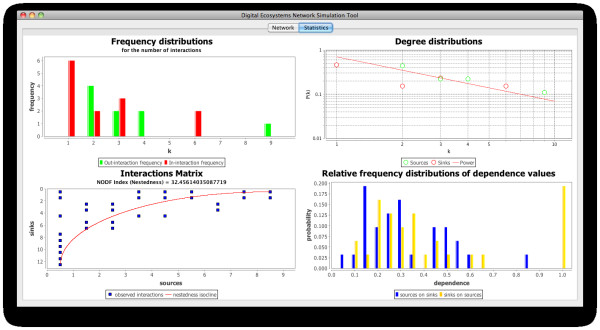
**Properties derived from the network of interactions in community number 3 in our simulations**. The community network is displayed in figure 9.

**Figure 12:**
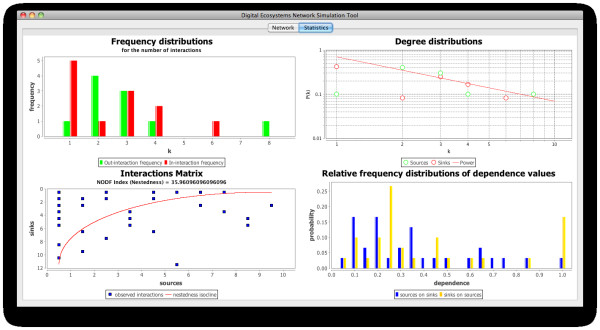
**Properties derived from the network of interactions in the OFLO natural community**. The community network is displayed in figure 10.

Natural communities, as we have seen above, are somewhat less predictable than our simulated ones. This can be confirmed when comparing the interactions matrices of our simulated community number 3 and the OFLO natural community (bottom left plots of Figures 11 and 12 respectively): community number three presents a much more organised structure, with few interactions below the isocline of perfect nestedness, while the OFLO community presents not only more interactions below the isocline but also a few of them are actually far removed from it. Also the degree distributions in both communities, although agreeing in the overall pattern of power law fit, differ subtly by the fact that the values in our simulated community are closer to the fitted line (top right plots in the figures).

In spite of these differences, the communities present very similar distributions of the frequencies of the number of interactions (top left plots), with practically all the nodes possessing less than five interactions and the majority of them with two or one. Additionally, only three nodes in the case of community number 3 and two in the case of the OFLO community possess more than five interactions: reaffirming in this way the scale-free character displayed not only by the natural communities employed here as references, which is expected from this type of networks, but also by our simulated communities as an emergent feature of self-organisation in our agent-based simulations.

In the bottom right plots of Figures 11 and 12 we can observe the distribution of the dependence values (how dependent is a species on the others) for the simulated and the natural communities respectively. Again, there can be seen the similarities between these two instances of artificial and natural communities, with the majority of the dependence values located between 0.0 and 0.4 (agents/species not very dependent on other agents/species) and few values above this range. It is important to realise however that the probability of a dependence value of 1.0 (the highest value), which implies that there may exist a node which is entirely dependent on another, is high for the dependence of visitor on host agents, in comparison with the other values of dependence; and this happens in both the natural and the artificial community. This might be due to the fact that a considerable number of visitor species on the network are involved in only one interaction and this could potentially mean that, in the event of losing the resource they exploit and if they are not able to adapt to exploit a different one, they could fail to survive, becoming in this way part of the cascading effects of an extinction.

**Figure 13:**
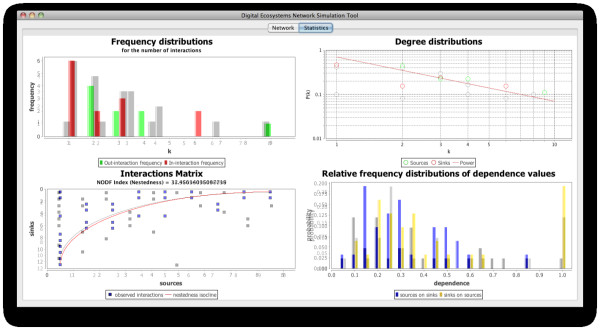
**Properties of the communities number 3 (Figure 11) and OFLO (Figure 12) overlaid**. In this plot we can see Figures 11 and 12 overlaid on each other, which facilitates the comparison between the properties of the community number 3 and the OFLO community. The datasets presented in grey in each of the plots correspond to the OFLO natural community, while the plots shown in their original colour correspond to community number 3.

Figure 13 shows the plots displaying the properties of community number 3 (Figure 11) and the OFLO natural community (Figure 12) overlaid, which facilitates the comparison between the datasets. Data shown in grey on the plots corresponds to the OFLO natural system while data in colour comes from the simulated community 3. In this picture the similarities and differences between these communities, as highlighted above, become more obvious and it can be seen how the interactions and dependence values distributions follow similar patterns, with slight differences; and how the interaction matrix for community number 3 is better organised, from the point of view of the nestedness isocline, than its counterpart in the OFLO community (grey squares in the bottom left plot of Figure 13).

The similarities found between natural communities and our simulated ones, not only in terms of the network structure, but also in terms of their features and characteristics, let us see how the mechanisms implemented at the individual interaction level between artificial agents has allowed us to obtain system level properties that are commonly encountered in natural communities. This model can thus help us study and analyse the possible mechanisms by which these properties, and the features of stability and robustness they provide to natural ecosystems emerge; not only by employing the example interaction protocol we have used for demonstrating the viability of this approach, but also by incorporating to the model many other mechanisms and processes that could in theory provide these systems with the characteristics they display and that are awaiting to be experimentally tested.

## Conclusions

In this paper we have presented an automated method for experimentation in ecological theory. The experiments that can be performed using this approach are based on an agent-based model that is able to obtain the network of interactions among simulated species in an artificial community that interact based on a protocol of interaction which relies heavily upon ecological theory for the representation of the relationships among species as they occur in real communities. The ultimate goal of this model is the analyses of the possible mechanisms and processes by which the characteristics and patterns observed in the network of interactions among species in natural communities, and that are believed to account for certain features such as stability and robustness in these ecological systems, arise.

The emergence of topological patterns in ecological networks has puzzled researchers in theoretical ecology, and many processes have been proposed as explanations for this emergence. In this work we take as an example the mutualistic relationship between plants and their animal pollinators to design an interaction protocol based on mechanisms believed to be involved in this kind of interactions to demonstrate that, to some extent, interesting community patterns, similar in many ways to the kinds found in this type of natural communities, are found only by enforcing interaction models thus engineered between agents in the agent-based model.

An automated framework of this kind, that allows ecologists to experiment with different mechanisms in order to find the ones that are most likely involved in the development of the intricate networks of relationships we find in nature, can enhance the experimental capabilities of researchers in this field, contributing in this way to the advancement of the understanding of the mechanisms behind community organisation in natural systems, and ultimately ecological theory.

In spite of the advantages mentioned above, the model presented here is limited by the fact that we are only considering mutualistic interactions. Our framework will thus benefit from a broader view on the community by including other kind of ecological relationships such as predator-prey and parasitic interactions. Although the current state of model may be useful for the study of mutualistic interactions, and any researcher might be able extend this only by specifying new interaction protocols, an interesting venue to continue its development is to perform a study similar to that presented here on the mechanisms behind mutualistic interactions but on other types of relationships, which will allow us to define new kinds of interactions and analyse the outcomes of the extended model.

Although the model works well from a behavioural point of view, it is not able to relate interactions to a phylogenetic background of species, which has been recently studied as a factor that could contribute to the appearance and maintenance of interactions between certain species [31]. Including a genetic and evolutionary component to this framework might be of interest not only for this purpose but also to study the role of evolution and coevolution in community organisation.

Another interesting venue for further development is the adaptation of this generic model to an specific mutualistic system: by changing some sections of the interaction protocol or the way agents behave we can match more closely our model to the studied system in order to study their particularities.

## Methods

We specify an ecologically inspired interaction model (IM) between agents in a agent-based model for the study of ecological interactions among species in natural communities. The simulated ecosystem enabled in this way is implemented using the OpenKnowledge system and therefore, LCC for the specification of the IM (as introduced in the Background section). In this section we describe the design and implementation of this digital environment, with particular emphasis on the ecological concepts and mechanisms used as the source of inspiration for defining the ecological protocol.

### An Ecologically Inspired Interaction Protocol

For describing interactions among agents in our simulated ecosystem we take inspiration from mechanisms believed to account for system-level patterns in natural communities [20], specifically we have employed some concepts that are related to the processes of *trait matching and spatial distribution in naturally *occurring populations:

1. **Traits**: a set of characteristics that define any entity within the system.

2. **Degree of complementarity**: the degree to which any trait of a given individual/agent is complementary to another trait possessed by another agent within the ecosystem.

3. **Habitat**: a representation of the part of the environment where the agent spends most of its live. An agent is more likely to interact with others in its same habitat.

4. **Meta-communities**: aggregation of entities that belong to different habitats (or regions) and that occasionally interact when certain conditions are met.

5. **Niche**: the multidimensional space composed by different characteristics of the environment (relationships) in which a given agent lives. This is an emergent property, and will, in our case, depend on the composition of the community, i.e. the agents with which interaction is feasible.

6. **Fitness**: a measure that is used to determine how good an agent is doing during its lifetime in the ecosystem.

These features, sometimes employed to characterise species in natural communities, will help us not only to define interactions between our agents, but also to design the internal structure of the agents themselves for coping with their interaction partners.

**Figure 14:**
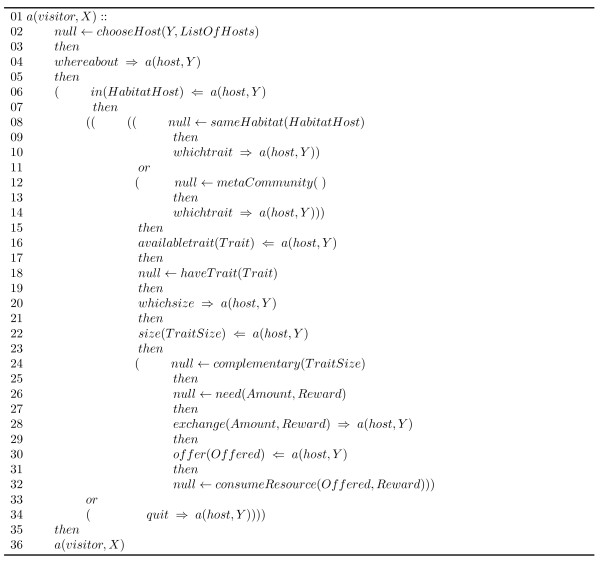
**Ecologically inspired interaction model, written in LCC, for agents coordination in an artificial ecosystem**. The "visitor" role is specified in this section of the protocol.

**Figure 15:**
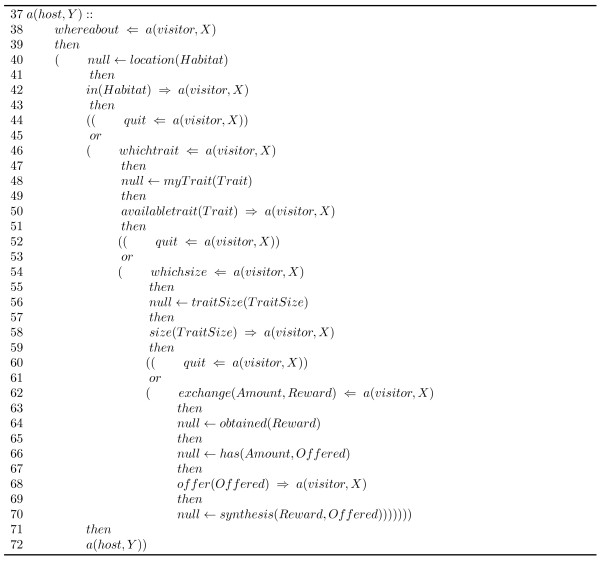
**Ecologically inspired interaction model, written in LCC, for agents coordination in an artificial ecosystem**. The "host" role is specified in this section of the protocol.

Figures 14 and 15 show the IM, written in LCC and based on the ecological concepts described above, that specifies the interactions between agents in our simulated ecosystem.

Where, as introduced in the Background section (*LCC Syntax *), *null *denotes an event which does not involve message passing; the operator :: is used to declare the definition of a role within the protocol; and the operators ←, *then *and *or *are connectives for logical implication, sequence and choice respectively. *M *⇒ *A *denotes that a message, *M*, is sent out to agent *A*. *M *⇐ *A *denotes that a message, *M*, from agent *A *is received.

In our example we have defined the roles of "host" and "visitor" in resemblance to the actors taking part in an ecological mutualistic interaction. We can see how the ecological concepts introduced above of: traits, degree of complementarity, habitat, and meta-communities; are explicitly introduced in the interaction protocol specification.

In any given execution flow of this protocol the agent acting as visitor will initiate the interaction by searching for appropriate partners (line 2 in the protocol); for this action the *chooseHost *predicate is used for selecting one of the available hosts in *ListOfHosts*. The selected host is referenced by the variable *Y*. Once it finds a suitable host, the interaction develops through the exchange of a series of messages where the agents exchange information about:

1. The habitat they are inhabiting and whether they will form a meta-community (lines 4-8,12 and 38-42). In this part of the interaction the visitor agent sends the *whereabout *message to the host in order to determine its habitat location, and consequently, the latter responds using the *in *message which contains a reference to the habitat (*Habitat, HabitatHost*) in which it is located. The host obtains a reference to its habitat employing the *location *constraint. The visitor agent then checks whether it shares habitat with the host using the *sameHabitat *predicate; if they do not share the same habitat, the visitor agent can decide whether to form a meta-community with the host agent using the *metaCommunity *constraint.

2. The matching trait for the interaction (lines 10,14-18 and 46-50). This step is performed using the *whichtrait *message, sent by the visitor agent to the host in order to find out the trait of interest. The host solves the constraint *myTrait*, and sends its *Trait *to the visitor through the *availableTrait *message. The visitor then confirms whether it can cope with that *Trait *using the *haveTrait *predicate.

3. Its degree of complementarity (lines 20-24 and 54-58). The information about the degree to which the matching trait is complementary is exchanged using the *whichsize *and *size *messages. The host uses the *traitSize *constraint to determine a value (*TraitSize*) used to evaluate the trait compatibility, which is finally evaluated by the visitor through the *complementary *constraint.

4. And the resource amount they will exchange (lines 26-32 and 62-70). Here the messages *exchange *and *offer *are employed to exchange information about the *Amount *of resource required by the visitor and the *Reward *offered to the host. The host assimilates the *Reward *using the *obtained *constraint and determines the amount of resource (*Offered*) it is able to offer through the has predicate. Finally, the exchanged resources are consumed using the *consumeResource *and *synthesis *constraints by the visitor and host agents respectively.

The *quit *message (lines 34, 44, 52, and 60 in the protocol) is used by the agents to terminate the interaction whenever is found that the necessary ecological conditions for it to happen are not met.

The concepts of niche and fitness are embedded within each of the agents themselves and represent the set of partners that can interact with a particular agent based on the IM, and a measure of how well the agent is performing, respectively.

### Agents in the OpenKnowledge System

In order to be able to take part in interactions described by the protocol introduced above, agents need to implement the constraints specified by each of the roles they want to take during any given interaction. This is done through the implementation of OpenKnowledge Components (OKCs) (see *OpenKnowledge *in the Background section). Since the OpenKnowledge system is implemented in the Java programming language [33], the OKCs are merely java classes that implement, in the form of methods, the constraints embedded in the protocol.

We implemented the OKCs that will allow our agents to interact by enacting the IM, using ecological concepts as those employed for the specification of the interaction protocol. Each constraint solved by the agents instantiating the OKCs has an ecological meaning, and jointly they are meant to promote mutualistic behaviour between the agents composing the digital environment.

The concept of fitness, as introduced above, is part of the agent itself, and is included in the agent's implementation by specifying a minimum amount of resources that must be maintained for the agent to be able to survive. The survival ability of a given agent is then, as it is in its natural counterparts (i.e. organisms in natural communities), directly correlated with its efficiency within the system, determining in this way its fitness measure.

The resolution of the constraints is determined in each agent by a series of attributes that are part of their specification and represent the knowledge they possess about the environment in which they live, as well as their internal properties. Some of these attributes, such as: the habitat they occupy, the ability they have to form meta-communities, the traits they possess and their features, the amount of resource they are willing to offer, and the amount they need to obtain from their partner; will define the niche that a given agent occupies, since these will restrict in an unique manner the set of other agents present in the environment with which it will be able to interact.

It is important to note at this point that the features regarding the traits possessed by each agent are measured using an index of complementarity, which has been named *size *within the protocol in resemblance to the feature that is more commonly used in nature to determine the complementarity between traits of different individuals (e.g. the size of the bill of a hummingbird against the size of the corolla of the flower it aims to pollinate).

#### The Visitor OKC

The visitor OKC implements the following methods, as specified by the IM:

• chooseHost: this method is used by the agent to select a host to interact with from the list of available hosts. The OpenKnowledge offers the functionality of locating all the peers that are subscribed to a particular role. The chooseHost function takes the list of peers (agents) that are subscribed to the host role and selects one for interaction.

• sameHabitat: this constraint is used by the agent to determine whether it currently occupies the same habitat as the host with which it intends to interact. The parameter *HabitatHost *is received by this method which, by performing a simple number comparison (the habitat is represented by a number within the agent), returns true if the visitor agent is in the same habitat as the host or false otherwise.

• metaCommunity: agents are able to form meta-communities by interacting with other agents that are not in their same habitat. For solving this constraint each agent possesses a value that determines the probability with which it will interact with an individual in other habitat. Individuals forming meta-communities will spend more resources in the interaction than if they were to interact with others in the same habitat, for this reason, this kind of interactions (as in nature) occur rarely. This method implements thus, the process of deciding whether to interact with an agent belonging to a different community.

• haveTrait: this function is employed by agents taking the visitor role for establishing whether they possess a certain characteristic. In our system there are finite number of characteristics, and each agent can possess any combination of them. When an agent is trying to interact with another they must agree on a trait that allows them to create a relationship link. The method receives the parameter *Trait *and will return true if the agent possesses it and false otherwise.

• complementary: after asking the agent with the host role the "size" of its trait (i.e. the measure that allows to compare traits in order to determine the degree to which they are complementary), the OKC allows to establish whether the selected trait is complementary with the matching trait in the other agent using this function.

• need: using this function the visitor agent is able to calculate the amount of resource it will demand from the host agent, value that is stored in the *Amount *parameter, and the amount of it that is going to be offered to the host as a *Reward*. Both of this quantities are calculated based on the complementarity of traits.

• consumeResource: this method allows for the update of the resource that the visitor agent has based on the *Offered *amount received from the host and the *Reward *offered to it during the negotiation process. Also, the implementation takes into account resources spent in other activities or living costs and these factors are added to the calculation of the final resource kept by the agent.

It is important to note here that a value indicating the degree of complementarity between the trait of a given agent and that of its interacting partner, which is calculated based on the difference between the sizes of the trait being considered, is stored within the agent taking the visitor role and is used for determining the reward and the amount of resource gained.

This *complementarityDegree *is then employed by the visitor agent to calculate the percentage of resource it will demand from its host and the amount of reward that it will offer to it. The more the complementary their traits are, the more resource the visitor will be able to demand and the more the reward it will be in a position to offer. This again is generally the rule in natural interactions, where morphological constraints, and the degree to which they match amongst interacting partners, determine the amount of resource they are able to exchange, and therefore, the likeliness of that interaction to happen.

#### The Host OKC

In order to be able to take the "host" role in an interaction described by the IM introduced above, an agent must instantiate an OKC implementing the following methods:

• location: using this method, an agent is able to retrieve the habitat it is inhabiting in any given moment, which is maintained as a variable of the agent, and to assign its value to the parameter *Habitat *that is given as an input to this function. This value will be sent to the visitor agent for determining whether the agents will successfully complete the interaction, as explained above.

• myTrait: after sending the information about the habitat it occupies, the agent taking the role of host will be asked to provide information about the trait available for the current interaction. Using the *myTrait *method, it will recover this information from its internal state and will pass it to the visitor agent using the *Trait *variable.

• traitSize: this function is used to provide the value of the size of the trait being considered (stored in the *TraitSize *variable), and which will be sent to the visitor agent which in turn will calculate the complementarity degree as previously explained.

• obtained: through this method, the host OKC obtains the value of the *Reward *offered by the visitor in this interaction and stores it as part of the host's internal state.

• has: this function receives the *Amount *of resource demanded by the visitor agent; and based on this, the internal resources of the own host agent, and the reward offered, calculates the amount of resource that it will provide to the visitor and stores it in the variable *Offered*, that will be sent to that agent.

• synthesis: taking into consideration the *Reward *obtained from the visitor and the resource *Offered *during the interaction, this method will update the amount of resource available to the host after the interaction is completed.

The OKCs thus implemented are instantiated by peers in the OpenKnowledge system, that represent the agents in our simulated ecosystem. This process allows peers to take the roles specified within the IM and that are realised by the OKCs instances.

During the initialisation process, every agent is assigned the role it is going to take in any instance of the IM. Through the discovery service provided by the OpenKnowledge platform, the IM is published and all the peers in the network are then able to take part on interactions defined by it, as long as they have the necessary roles enabled.

When the system finds suitable partners for an interaction, these agents will be "recruited" and they will instantiate the OKC corresponding to the role they are about to fulfil within the interaction. The execution of the interaction protocol then proceeds and the constraints composing it are solved by each agent using the respective OKC instances, this process determines the path of the interaction (remember that agents can dismiss an interaction based on certain conditions) and the flow of messages exchanged as part of it. Upon successful completion of the interaction, agents are then freed to engage in another one with any other suitable agent on the network.

### Experimental Design

Our model has a number of parameters and settings that can be configured in order to produce different outcomes given different circumstances for the agents to interact. Our default general settings however, define certain values that are common to all of the simulation results presented in the Results section. The main settings concerning the model are:

1. interaction protocol: the interaction protocol employed is the one presented above, and this is the way agents interact, however, as also explained in that section, the protocol provides the possibility for agents to leave interactions at different points of their execution based on their capacities and necessities, and therefore, the outcome of any given interaction may differ from another one.

2. agent types: even though agents in our digital ecosystem can potentially take any of the roles specified in the interaction protocol, we have constrained agents to take only one of the roles available, in order to facilitate the analysis of the interactions by closely resembling the structure of mutualistic networks in nature. This does not affect the results obtained and they will still apply even if agents take both roles in the interaction (ideally of course when the other role is taken by another agent).

3. number of agents: in resemblance to the proportion of plants versus pollinators in plant-animal interaction networks, where there are generally more plant species than animal ones, we set the number of host agents in our digital ecosystems to ten and that of the visitors to fifteen. Although the proportions of each type of agent in relation with each other are taken from natural communities, the actual number in each category is somewhat arbitrary, but it is a way to keep the system within a manageable size for the execution of the experiments. It is important to note here that even with the number of agents set to a particular value, there might be agents that for some reason, like for example the lack of resources, are not able to embark upon any interactions; in these cases, these agents are not presented in the graph representation of the network of interactions.

4. number of habitats: the habitat a given agent occupies will partially determine whether it will be able to interact with the agent selected for interaction; they will be more likely to interact if in the same habitat than otherwise. In our experiments the default number of habitats employed is two, and agents are evenly distributed among both habitats.

5. minimum and maximum resources: any given interaction, as seen above, is based around the exchange of resources that will ultimately allow agents to survive; at the beginning of each run, agents are given a certain amount of resources that is selected randomly from a normal distribution bounded by a minimum and maximum amount of resources. By default the range for this amount is between five (min resource) and twenty (max resource). Although the initial amount of resource may determine the fate of an agent, these numbers were selected in order to avoid any premature agent death due to lack of resources.

6. meta-community formation: agents possess the ability of interacting with individuals belonging to different habitats than their own, when this happens they are said to form a meta-community. In our simulations, agents are only allowed to take part in a meta-community interaction if their own resources are at least half the maximum amount of resources (previous point), because if they get involved in this kind of interactions they will loose more resources than if interacting with individuals in the same habitat. The probability of any given agent forming a meta-community association is 0.1.

7. maximum trait size: as introduced in the protocol specification, the size of a trait will determine the degree to which agents in an interaction are complementary to one another. In our model we have a parameter specifying the maximum size of the interaction trait, and all the agents will have sizes ranging from zero to this maximum size. The default value for the max size of traits in our system is five.

8. number of interactions: an interaction is considered to be successfully completed when the partners involved in it actually exchange resources, i.e. all the ecological conditions set by the protocol are met and therefore no agent has quit the interaction prematurely. Each one of our simulation runs were allowed to evolve for a fixed number of successfully completed interactions: three hundred (300). This number was selected based on the observation of previous runs in which the network of interactions reached an stable state, in the sense that its topological configuration was not noticeably affected by further interactions, as the number of interactions amongst the agents in the system approached this number. This has no particular ecological meaning since it is only an artifactual consequence of the model in which the agents have originally no interactions with others. It is difficult to compare this to any situation in the real world, because in a real ecosystem, the interactions network is already formed and the stability of the system is evaluated from the point of view of its resilience rather than its time to achieve a particular form, which occurs at much larger timescales and is a much more dynamic process in terms of species composition.

Among the parameters introduced above we find some ecological, like: the agent types, the number of habitats and the meta-community formation probability; which default values have been taken from values found in ecological systems for this kind of features. Whereas the default values for parameters such as the minimum and maximum amount of resources within the agents, or the maximum trait size where arbitrarily defined in order to closely simulate the conditions that will commonly determine the outcomes of interactions among species in the type of natural systems subject of this study.

In any case, and in order to evaluate the impact of changing some of these parameters in different ways, we performed a series of sensitivity analyses with respect to the initial values of these variables [34], and we found that they were robust to changes and the organisation of the communities arising from the simulations performed were not noticeably affected by changes on their values.

The experimental conditions described above provide the configuration for our framework, on top of which we run a series of simulations that allowed us to evaluate our model in a controlled and methodological way and obtain the results presented in the Results section. In every simulation execution we deploy the OpenKnowledge platform and create every agent following the specifications outlined above; we allow for the interactions among agents based on random encounters between them and after the number of interactions specified as a termination criterion (three hundred) has been reached, the simulation finishes and the final network of interactions can be analysed.

### Network Metrics

Having the representation of the network of interactions in the form of a graph allows us to extract information from it and to calculate interesting properties that can account for patterns displayed by the network. For the analysis of the interactions networks obtained from the simulated communities we have taken four properties that are commonly used in the field of mutualistic networks to analyse this kind of webs of interactions. These are presented in the results above since they help us study our agents networks:

1. frequency distribution of the number of interactions: this represents the distribution of the frequencies with which we encounter a node with a certain number of interactions. It is displayed in the form of a plot which allows to obtain a figure of the commonness of well connected versus less connected nodes.

2. degree distribution: the degree of a node is the number of links it has; in our case, since the edges connecting nodes are directed (arcs) we will have in-degree (the number of edges in which the node is the sink or head) and out-degree (the number of edges in which the node is the source or tail). The distribution of the frequencies of degrees is a good indicative of the extent to which a given network possesses scale-free features.

3. interactions matrix and nestedness index: the interactions matrix let us see the relations happening between pairs of agents. Based on these interactions we calculate the isocline of perfect nestedness, as introduced in [35], which by taking into account the number of interactions occurring in the network obtains an estimate of a perfectly nested matrix and gives a curve that allows us to visualise the pattern that we should expect from it; this curve, plotted on top of the actual interactions matrix gives an idea about how nested our matrix is. Additionally, we calculate the Nestedness metric based on Overlap and Decreasing Fill (NODF), as presented in [32], and which is a metric commonly used in the analysis of nestedness in ecological networks [36] for determining the extend to which a given interaction network presents a nested pattern.

4. relative frequency distribution of dependence values: this plot displays the distribution of the frequencies of the values of dependence of one node on another; we have dependence of host nodes on visitor nodes and can also calculate the dependence of a visitor agent on a host, displaying in this way the extent to which agents are dependent on others for survival. This plot is complementary to the distribution of the frequency of the number of interactions in the sense that we can extract information about the average number of links per node from the latter and get an idea of how strong links may be within the network from the dependence values.

## Competing interests

The authors declare that they have no competing interests.

## Authors' contributions

ML developed the ecological ideas for the specification of the interaction protocol presented. He implemented the simulation model that allows the specification of protocols for experimentation and the use of network measurements for the analysis of the results. DR designed the LCC language used to specify interactions in the simulator and was an architect of the OpenKnowledge system. He assisted ML in expressing ecological relationships in the form of LCC interaction protocols. Both authors have read and approved the final manuscript.
